# *flaA*-SVR Based Genetic Diversity of Multiresistant *Campylobacter jejuni* Isolated From Chickens and Humans

**DOI:** 10.3389/fmicb.2019.01176

**Published:** 2019-05-28

**Authors:** Kinga Wieczorek, Tomasz Wołkowicz, Jacek Osek

**Affiliations:** ^1^Department of Hygiene of Food of Animal Origin, National Veterinary Research Institute, Pulawy, Poland; ^2^Department of Bacteriology and Biocontamination Control, National Institute of Public Health – National Institute of Hygiene, Warsaw, Poland

**Keywords:** *Campylobacter jejuni*, chicken food chain, humans, antimicrobial resistance, *flaA*-SVR sequencing

## Abstract

*Campylobacter jejuni* is one of the most common causes of human foodborne bacterial infections worldwide. The objective of this study was to assess the molecular diversity, using *flaA* sequencing, of 602 *C. jejuni* isolated from chicken food chain, i.e., chicken feces (*n* = 151), chicken carcasses (*n* = 150), chicken meat (*n* = 150), and from humans (*n* = 151) and to determine antimicrobial multiresistant profiles of the isolates as well as to analyze the relationship of the isolate genotypes with their antimicrobial resistance profiles and source of isolation. Multidrug resistant patterns were identified in 110 (18.3%) *C. jejuni* isolates recovered from all sources and most isolates were resistant to ciprofloxacin (CIP), nalidixic acid (NAL), streptomycin (STR), and tetracycline (TET) (92; 15.3%) or ciprofloxacin, streptomycin, and tetracycline (13; 2.2%). Only a few isolates were multiresistant to ciprofloxacin, nalidixic acid, tetracycline, and erythromycin (3; 0.5%) or ciprofloxacin, nalidixic acid, streptomycin, tetracycline, and erythromycin (2; 0.3%). A total of 79 *flaA*-SVR subtypes were identified, including 40 (50.6%) unique to the isolates’ origins, with the most common sequence types 16, 54, 36, 34, and 287 which covered 56 (9.3%), 50 (8.3%), 48 (8.0%), 35 (5.8%), and 32 (5.3%) of *C. jejuni* isolates, respectively. It was found that 13 isolates had the novel *flaA*-SVR subtypes which were not present in the pubMLST database. These isolates were recovered from chicken feces (6 isolates), carcasses (2 isolates), meat (one isolate) and from humans (4 isolates). Multiresistant *C. jejuni* were classified into 26 different sequence subtypes. Among the most numerous multidrug resistant profile CIP+NAL+STR+TET 21 different *flaA*-SVR subtypes, with total of 92 isolates, were identified. Most of them were classified to 287 (18; 19.6% isolates), 100 (13; 14.1%), 34 (9; 9.8%), 208 (8; 8.7%), and 781 (8; 8.7%) molecular variants. Isolates resistant to CIP, STR and TET (13 isolates) were mainly from chicken feces (12 isolates) and classified into 5 *flaA*-SVR sequence types, with the most common 36 (8 isolates). The obtained results show a broad molecular diversity of multiresistant *C. jejuni* isolates and suggest chickens as a possible source of human *Campylobacter* infections in Poland.

## Introduction

*Campylobacter*, especially *Campylobacter jejuni*, is one of the most common causes of foodborne bacterial infections worldwide (Allos, 2001; Bolton, 2015; Kaakoush et al., 2015; Tresse et al., 2017). Campylobacteriosis is also the most commonly reported zoonosis in the European Union with 246,158 confirmed cases and a notification rate of 64.8 per 100,000 population in 2017 (European Food Safety Authority [EFSA] and European Centre for Disease Prevention and Control [ECDC], 2018). However, it has been estimated that the real number of *Campylobacter* infections occurring yearly may be several millions and the annual cost of the disease is almost one billion of United States dollars (Havelaar et al., 2009; Silva et al., 2011; Kaakoush et al., 2015). Several studies showed that one of the most important transmission routes of *C. jejuni* to humans is handling, preparation and consumption of contaminated food of poultry origin (Allos, 2001; Park, 2002; Humphrey et al., 2007). *C. jejuni* colonizes chicken intestines at the number of 10^8^ cells per gram of cecal contents or greater without causing disease (Beery et al., 1988; Sahin et al., 2002). After colonization of the first birds in a flock, the bacteria rapidly spread throughout the flock and remain present until slaughtering (Wagenaar et al., 2006). *C. jejuni* colonizes the mucus mainly of the cecal epithelial cells and the small intestine but may also be found in other parts of the gut (Newell and Fearnley, 2003). Transmission from chickens to humans most commonly occurs through consumption and handling of chicken meat and meat products contaminated with these bacteria during slaughter and carcass processing (Kaakoush et al., 2015). It has been estimated that the chicken reservoir as a whole is estimated to be responsible for up to 80% of human campylobacteriosis cases (European Food Safety Authority [EFSA] and European Centre for Disease Prevention and Control [ECDC], 2018).

Most campylobacteriosis cases are usually self-limiting and do not require antimicrobial treatment. However, severe infections occasionally require antimicrobial therapy often with macrolides (erythromycin or azithromycin) and, to a lesser extent, with fluoroquinolones, tetracyclines, or gentamicin when infection becomes systemic (Iovine, 2013). A major concern with regard to treating campylobacteriosis in humans is antimicrobial resistance, particularly resistance of *C. jejuni* to fluoroquinolones and macrolides, which has increased significantly over the past two decades (Melero et al., 2012; Piccirillo et al., 2013; Wieczorek et al., 2013; Han et al., 2016; Mäesaar et al., 2016; Olkkola et al., 2016; Post et al., 2017; Woźniak-Biel et al., 2018). It has been suggested that food of animal origin, especially poultry meat, may represent a vehicle of transmission of resistant *Campylobacter* to humans (Aarestrup et al., 2008). Ciprofloxacin and erythromycin are the antimicrobials of choice for treatment of human campylobacteriosis (Ge et al., 2013; Iovine, 2013). The intensive use of antimicrobials in animals and in humans has led to an increase in the antibiotic-resistant *Campylobacter* population (Humphrey et al., 2007; Ge et al., 2013). Thus, monitoring of resistance of *C. jejuni* derived from infected patients and food of animal (poultry) origin is highly relevant to public health.

Molecular typing is an important tool for evaluation of diversity and transmission routes of *Campylobacter* isolates contaminating the food chain and isolated from patients with diarrhea.

Several studies of *C. jejuni* demonstrated that this microorganism is genetically diverse, predominantly as a result of frequent intra- and interspecies genetic recombination, within a weakly clonal population structure (Dingle et al., 2001; Suerbaum et al., 2001; Manning et al., 2003). In order to investigate the epidemiology of *C. jejuni*, molecular subtyping methods with enhanced discriminatory power are used (Wassenaar and Newell, 2000; Wieczorek et al., 2017). One of them is direct sequencing of PCR-amplified short variable regions (SVRs) products of the flagellin-encoding A (*flaA*) gene (Wassenaar et al., 1995; Harrington et al., 1997). It was shown that the SVR region is located between 450 and 600 base positions in the *C. jejuni* *flaA* encoding gene (Meinersmann et al., 1997). Several studies have demonstrated that direct sequencing of PCR-amplified short variable regions (SVRs) of the *A* gene is a useful tool for *Campylobacter* genotyping, offering similar or higher discriminatory power than multilocus sequence typing (MLST) and pulsed-field gel electrophoresis (PFGE) (Meinersmann et al., 1997; Wassenaar et al., 2009; Wirz et al., 2010; Magnússon et al., 2011; Gomes et al., 2016). Additionally, sequences of *flaA*-SVR nucleotide alleles are stored in the pubMLST database^1^, and allow open access to the *flaA*-SVR types of *Campylobacter* strains isolated around the world. Furthermore, it has been shown that *flaA*-typing provides sufficient discrimination for its use as a subtyping method for *C. jejuni* (Suerbaum et al., 2001; Manning et al., 2003). Despite the observation that recombination rates in *C. jejuni* may potentially have an adverse impact on the reliable interpretation of *flaA*-typing (Wassenaar et al., 1995; Harrington et al., 1997), several studies showed that in the majority of *C. jejuni* isolates some regions of the flagellin-encoding A gene are genetically stable over long periods and may be used for molecular typing and differentiation of the isolates (Burnens et al., 1995; Owen et al., 1995).

The objectives of the present study were: (i) to determine antimicrobial multiresistant profiles of a collection of *C. jejuni* isolates recovered from chicken food chain and from humans with diarrhea, (ii) to assess the genetic relatedness of these isolates using *flaA* sequencing, and (iii) to examine the relationship of the isolate genotypes with their antimicrobial resistance profiles and source of isolation.

## Materials and Methods

### Isolation of *C. jejuni*

Sampling and isolation of *C. jejuni* from the poultry food chain and from humans with diarrhea were performed as described previously (Wieczorek et al., 2018). The detailed information on all 602 isolates are included in the Supplementary Material (Supplementary Table S1). In case of chicken *C. jejuni* (a total of 451 isolates), the samples were collected during years 2010–2016 according to the monitoring plan prepared in the Polish National Reference Laboratory for *Campylobacter*. Intact ceca from 10 birds were taken after evisceration, the content was pooled and one loop-full (10 μl) of the material was streaked directly onto Karmali agar (*Campylobacter* Agar Base + *Campylobacter* Supplement; Oxoid, United Kingdom) and *Campylobacter* blood-free agar (Oxoid) with CCDA selective supplement (Oxoid) and incubated at 41.5°C ± 1°C for at least 48 h ± 2 h in a microaerobic atmosphere generated using a CampyGen kit (Oxoid). From each fecal sample one presumptive *Campylobacter* isolate was then confirmed by PCR as described previously (Wieczorek et al., 2013). Briefly, one bacterial colony was suspended in 1 ml of redistilled water and centrifuged at 13,000 ×*g* for 1 min. The pellet was resuspended in 100 μl of 10 mM Tris buffer (A&A Biotechnology, Gdańsk, Poland) and DNA was isolated with the Genomic – Mini kit according to the manufacturer’s instruction (A&A Biotechnology). The PCR was performed using the following program: 94°C for 5 min (initial denaturation) followed by 30 cycles: 94°C for 1 min, 58°C 2 min, 72°C for 1 min. The final extension step was performed at 72°C for 5 min. The used PCR primers amplified fragments of the *mapA* (specific for *C. jejuni*) and the *ceuE* (characteristic for *C. coli*) *Campylobacter* genes. The amplification of the *16S rRNA* gene fragment, presents in both *C. jejuni* and *C. coli*, which allowed to evaluate the PCR reaction and the bacterial DNA, was also performed. A total of 151 *C. jejuni* isolates from chicken feces were used in the present study. The detailed information on the chicken samples are included in the Supplementary Material (Supplementary Table S1).

The swab samples from the neck skin and the skin surface under the wings of chicken carcasses were collected directly after immersion chilling (0–4°C) but before further processing and immediately transported to the laboratory in Amies transport medium with charcoal (Medlab, Poland). *Campylobacter* bacteria were isolated as described (Wieczorek et al., 2013). Briefly, the swabs were placed in 5 ml of Bolton enrichment broth (Oxoid) supplemented with vancomycin, cefoperazone, trimethoprim, and amphotericin B and incubated as described for fecal samples. The cultures were then plated onto Karmali agar (Oxoid) and *Campylobacter* blood-free agar with CCDA selective supplement (Oxoid) and incubated under the same conditions. From each sample one presumptive *Campylobacter* isolate was confirmed using PCR as described previously (Wieczorek et al., 2013). A total of 150 *C. jejuni* from chicken carcasses were collected for the current investigation.

The *Campylobacter* isolates from chicken meat (*n* = 150) were recovered using the ISO 10272-1 standard and *C. jejuni* were confirmed with the PCR method as described for the chicken carcasses.

A total of 151 *C. jejuni* isolates were obtained from patients with diarrhea using standard culturing techniques. Rectal swabs were directly streaked onto mCCDA agar (Oxoid) and incubated at 41.5°C ± 1°C for 48 h ± 2 h under microaerobic conditions. Then, typical *Campylobacter* colonies were selected for further investigation using standard biochemical tests. *C. jejuni* was identified with PCR as described previously (Vandamme et al., 1997). All *C. jejuni* identified in patients with diarrhea were isolated by regional diagnostic laboratories located in five voivodeships (administrative regions of Poland) during years 2011–2016 and confirmed by PCR at the National Veterinary Research Institute in Pulawy. The detailed information on the human samples are included in the Supplementary Material (Supplementary Table S1). The authors declare that the study did not need any recommendation or approval of an ethics committee nor written consent from the people from whom *C. jejuni* were isolated.

Altogether, 602 *C. jejuni* were isolated and stored at -80°C until further analysis.

### Antimicrobial Resistance

A microbroth dilution method was used to establish the minimum inhibitory concentrations (MICs) of six antimicrobials (gentamicin [GEN], streptomycin [STR], erythromycin [ERY], ciprofloxacin [CIP], nalidixic acid [NAL], and tetracycline [TET]) to *C. jejuni* isolates using EUCAMP2 Sensititre^®^ custom susceptibility plates (Trek Diagnostics, United Kingdom). The dilution ranges and cut-off values are presented in Supplementary Table S2 (Wieczorek et al., 2018). The isolates were sub-cultured twice on Columbia agar (Oxoid) at 41.5°C for 48 h under microaerobic conditions. The minimum inhibitory concentration of the antimicrobial agents was determined using Mueller-Hinton broth (Oxoid) supplemented with 2–2.5% horse blood (Trek). The plates were incubated at 37°C for 48 h under microaerophilic conditions and read using the Vision^®^ system (Trek). The antimicrobials and cut off values used for the interpretation of the MIC results were in accordance with EUCAST (Sifré et al., 2015) and the European Union Reference Laboratory for Antimicrobial Resistance. Multidrug resistance of the isolated *C. jejuni* was defined as resistance to at least three classes of antimicrobials used in the study (Magiorakos et al., 2012).

### DNA Extraction and *flaA*-SVR Sequencing

One bacterial colony was suspended in 1 ml of sterile, DNase- and RNase-free water and centrifuged at 13,000 *g* for 1 min, and DNA was extracted using the Genomic-Mini kit (A&A Biotechnology, Poland) according to the manufacturer’s instruction. DNA was then utilized as a template in PCR with the forward 5′-ATGGGATTTCGTATTAACAC-3′ and reverse 5′-CTGTAGTAATCTTAAAACATTTTG-3′ primers (Wassenaar and Newell, 2000), using the following amplification conditions: initial DNA denaturation at 94°C for 2 min followed by 35 cycles of 94°C for 1 min, 50°C for 1 min and 72°C for 1 min. The final extension step was performed at 72°C for 1 min. Purification and sequencing of the amplified products was performed by an external company (Genomed, Warsaw, Poland) using the BigDye Terminator v. 3.1 kit (Applied Biosystems, United States). The sequencing products were separated in a 3730 × l DNA Analyzer capillary sequencer (Applied Biosystems) and the DNA sequences were then imported and checked for quality using the BioNumerics v. 7.6 software (Applied Maths, Sint-Martens-Latem, Belgium). The sequences were then assigned allelic numbers based on the data present in the *Campylobacter* *flaA*-nucleotide database using sequence query *Campylobacter* locus/sequence definitions (see text footnote 1). If exact match was identified the number was assigned to the isolates. When any mismatches of DNA sequences to those present in the database were found, which suggested possible new *flaA*-SVR alleles, the isolates were sequenced once again and then submitted to the database administrator for confirmation. The sequences of all *C. jejuni* isolates examined in the present study are now present in the mentioned above database and could be identified by the number of *flaA*-SVR sequence type.

### Statistical Analysis

The chi-squared test with Yates’ correction was used to examine differences in the prevalence of *flaA*-SVR subtypes among *C. jejuni* isolated from different sources and *P* < 0.05 was considered significant. The genetic diversity of *C. jejuni* within the populations of isolates recovered from different sources was assessed by Simpson’s diversity index (ID) as described previously (Hunter and Gaston, 1988) using the online tool “Comparing Partitions” from the website http://www.comparingpartitions.info (Carriço et al., 2006). The data was directly transfer from the excel file to the online tool (Supplementary Table S3). The column corresponding to a different partition assignment and each value to a cluster identifier. The first row contains the columns titles. The proportional similarity index (PSI) was applied to compare sequence types distribution among *C. jejuni* isolates from various sources (Hunter and Gaston, 1988; Garrett et al., 2007). The frequency distributions of the different sources were estimated by calculating their similarity using the following equation: $PSI=1-0.5\Sigma_{i}\;\text{|}p_{i}-q_{i}\text{|}=\Sigma_{i}\;min\;(p_{i},q_{i})$, where p_i_ and q_i_ are the proportion of isolates from group p and q, respectively, belonging to type i. PSI ranges from zero to one, where one indicates that two groups are identical and zero means they share no types. Around 95% confidence intervals (CI) were computed using bias-corrected and accelerated non-parametric bootstrap. Calculations were performed using R, ver. 3.1.3 and @RISK for Excel, ver. 6.0.1 (Palisade Co., Ithaca, NY, United States). An index greater than 0.90 is considered desirable if the typing results are to be interpreted with confidence (Hunter and Gaston, 1988).

## Results

### *flaA*-SVR Sequence Types

A total of 79 *flaA*-SVR subtypes were identified, including 40 (50.6%) sequences unique to the isolates’ origin, with 15 sequences found only in *C. jejuni* from chicken feces, 12 subtypes in isolates from chicken carcasses, 7 sequences in chicken meat, and 6 subtypes detected only in isolates recovered from humans. Additionally, 24 different *flaA*-SVR subtypes were found in *C. jejuni* from all sources which cover 76.2% (459 out of 602) isolates (Supplementary Table S1). The most common sequence types identified among all 602 isolates tested were 16, 54, 36, 34, and 287 which included 56 (9.3%), 50 (8.3%), 48 (8.0%), 35 (5.8%), and 32 (5.3%) of *C. jejuni* isolates, respectively (Table 1). Among isolates from the chicken food chain (*n* = 451), 50 sequence types were identified in *C. jejuni* from feces, 47 variants from carcasses, and 39 types from meat, respectively. Most of them were classified to 16, 54, and 36 variants (total 103 out of 451 isolates; 22.8%). In the human bacterial population (*n* = 151) 37 different *flaA*-SVR sequence alleles were detected, mainly belonging to subtypes 16, 54, and 14 (total 55; 36.4% isolates) (Table 1).

**Table 1:** Prevalence of the most numerous *flaA*-SVR sequence types in *C. jejuni* tested.

Source of isolates		*flaA*-SVR sequence allele and number of isolates in each sequence type																	
		16*	54	36	34	287	14	49	100	239	278	21	78	208	222	5	10	57	Other (No. of different alleles)
Chicken	Feces	9	14	13	12	8	3	10	7	1	5	2	1	3	4	0	4	2	53 (34)
	Carcasses	12	10	11	10	5	6	4	6	3	6	2	5	7	3	3	0	1	56 (31)
	Meat	12	5	17	4	12	5	5	6	14	2	6	0	1	2	6	7	7	39 (23)
Human		23	21	7	9	7	11	4	2	2	4	6	8	3	4	3	1	2	34 (20)
Total		56	50	48	35	32	25	23	21	20	17	16	14	14	13	12	12	12	182 (62)


*Other includes 62 different sequence types, counting from 9 to one isolate; among them are 13 isolates with novel *flaA*-SVR sequences. ^∗^Statistically significant differences between the presence of the following *flaA*-SVR sequences of *C. jejuni* isolates from different sources were identified: 54 from carcasses and humans (*P* < 0.005); 239 from feces and meat (*P* < 0.005), from carcasses and humans (*P* < 0.05), from meat and humans (*P* < 0.005); 78 from feces and humans (*P* < 0.05), from meat and humans (*P* < 0.05); 5 from feces and meat (*P* < 0.05); 10 from carcasses and meat (*P* < 0.05). In case of the remaining *flaA*-SVR sequences no statistically significant differences between sources of the isolates were detected.*

Distribution of the most prevalent *flaA*-SVR genotypes in relation to the sources of the isolates is shown in Table 2. Among *C. jejuni* from the chicken food chain, the most numerous subtypes were classified into sequence variants 36 (41; 9.1% isolates), 16 (33; 7.3% isolates), and 54 (29; 6.4% isolates), whereas human isolates mainly belonged to genotypes 16 (23; 15.2% isolates) and 54 (21 (13.9% isolates).

**Table 2:** Prevalence of *flaA*-SVR sequence types in *C. jejuni* isolated from different sources.

Chicken feces						
Sequence types	54	36		34	49	16		287	100	117		278	781	10		222	Other**
No. (%) of isolates*	14 (9.3)	13 (8.6)		12 (7.9)	10 (6.6)	9 (6.0)		8 (5.3)	7 (4.6)	5 (3.3)		5 (3.3)	5 (3.3)	4 (2.6)		4 (2.6)	55 (36.4)
**Chicken carcasses**						
Sequence types	16	36	34	54	208	14	100	278	78	161	287	410	22	49	67	265	Other**
No. (%) of isolates*	12 (8.0)	11 (7.3)	10 (6.7)	10 (6.7)	7 (4.7)	6 (4.0)	6 (4.0)	6 (4.0)	5 (3.3)	5 (3.3)	5 (3.3)	5 (3.3)	4 (2.7)	4 (2.7)	4 (2.7)	4 (2.7)	46 (30.7)
**Chicken meat**						
Sequence types	36	239	16	287	10	57		5	21	100	14	49	54	34	320	1424	Other**
No. (%) of isolates*	17 (11.3)	14 (9.3)	12 (8.0)	12 (8.0)	7 (4.7)	7 (4.7)		6 (4.0)	6 (4.0)	6 (4.0)	5 (3.3)	5 (3.3)	5 (3.3)	4 (2.7)	4 (2.7)	4 (2.7)	36 (24.0)
**Humans**						
Sequencetypes	16	54		14	34	78		36	287		21		49	222	278		Other**
No. (%) of isolates*	23 (15.2)	21 (13.9)		11 (7.3)	9 (6.0)	8 (5.3)		7 (4.6)	7 (4.6)		6 (4.0)		4 (2.6)	4 (2.6)	4 (2.6)		47 (31.1)


*More than 2% of isolates belonging to each sequence types are shown. **Other includes sequences with equal or less than 2% of isolates as shown in Supplementary Table S1.

It was also found that 13 isolates had an *flaA*-SVR subtype which was not present in the pubMLST database. These isolates were recovered from chicken feces (6 isolates with the new sequences 1662, 1663, 1666, 1667, 1669, and 1673), chicken carcasses (2 isolates with the sequences 1670 and 1672), chicken meat (1 isolate with the sequence 1674), and humans origin (4 strains with the sequences 1664, 1665, 1668, and 1671). All these novel alleles were submitted to pubMLST database.

Overall, the *flaA*-SVR typing method was highly discriminative for all *C. jejuni* used in the study since the Simpson’s diversity index (D) achieved value 0.968, indicating considerable diversity in the bacterial population tested, although isolates collected from the chicken food chain displayed a higher genetic diversity than isolates from humans (Table 3). Taking into account the number of the *flaA*-SVR sequences, no significant difference of diversity was observed between isolates recovered from chicken feces, carcasses, and meat. The lowest genetic diversity was identified among *C. jejuni* isolates with multidrug resistance profiles, although the number of such isolates was lower than the total number of campylobacters identified in each tested group.

**Table 3:** Simpson’s diversity (DI) and proportional similarity (PSI) indexes of *flaA*-SVR sequencing within *C. jejuni* isolates from different sources.

Type of *C. jejuni* isolates (No. of isolates)		No. of *flaA*-SVR sequences	DI (95% CI*)	PSI (95% CI)			
				Chicken			Human
				Feces	Carcasses	Meat	
Chicken	Feces (*n* = 151)	50	0.962 (0.952–0.972)	1**	0.849 (0.815–0.885)	0.856 (0.822–0.889)	0.859 (0.824–0.891)
	Carcasses (*n* = 150)	47	0.968 (0.960–0.976)	0.849 (0.812–0.882)	1	0.856 (0.823–0.887)	0.860 (0.825–0.892)
	Meat (*n* = 150)	39	0.955 (0.943–0.966)	0.856 (0.822–0.888)	0.855 (0.820–0.886)	1	0.867 (0.835–0.901)
Human (*n* = 151)		37	0.940 (0.922–0.957)	0.860 (0.821–0.891)	0.862 (0.822–0.895)	0.868 (0.832–0.901)	1
Total (*n* = 602)		79	0.961 (0.956–0.966)	NA***			
Multiresistant (*n* = 110)		26	0.922 (0.902–0.942)	NA***			


*CI, confidence intervals with 95% confidence level. **1 = maximal similarity; 0 = maximal difference. ***Not applicable.

The PSIs were calculated to assess the similarity of *flaA*-SVR sequences distributions between different *C. jejuni* sources, i.e., humans and three stages of chicken food chain, i.e., feces, carcasses, and meat (Table 3). The *flaA*-SVR subtypes identified in the chicken samples were highly similar (PSIs above 0.8) and the similarity of the chicken and human isolates was also calculated at the comparable levels.

### Antimicrobial Resistance

The results of antimicrobial resistance of the *C. jejuni* showed that most of the isolates were resistant to ciprofloxacin (total 556; 92.4% isolates), nalidixic acid (538; 89.4%) and, to a lesser extent, tetracycline (412; 68.4%). Isolates from the chicken food chain were more often resistant to CIP than those from human patients. A similar relationship was observed for TET where the isolates from chicken feces were more often resistant than *C. jejuni* of carcasses and meat origin. A low number of isolates, irrespective of the origin, were resistant to STR (111; 18.4%). It was also found that only 5 of 624 isolates (0.8%) displayed resistance to ERY and all of them were recovered from the chicken food chain.

Multiresistance patterns were identified among 110 out of 602 (18.3%) *C. jejuni* isolated from all sources (Table 4). The vast majority of such isolates were resistant to CIP, NAL, STR, and TET (92 out of 110 isolates; 83.6%) and they were mainly recovered from the chicken food chain (80; 72.7% isolates). Detailed information on antimicrobial resistance of each *C. jejuni* isolate tested in the study, including the minimum inhibitory concentrations (MICs), is shown in Supplementary Table S1.

**Table 4:** Relationship between multiresistance and *flaA*-SVR sequence types in *C. jejuni* tested.

**Antimicrobial resistance pattern (No. of isolates)**	**Source of isolates**		**No. of isolates with *flaA*-SVR sequence allele**																									
			**5**	**14**	**16**	**18**	**21**	**22**	**34**	**36**	**54**	**67**	**100**	**105**	**121**	**136**	**208**	**265**	**269**	**278**	**287**	**410**	**423**	**781**	**1523**	**1556**	1662*	1663*
CIP+NAL+STR+TET (*n* = 92)	Chicken	feces							5				6			1	1	2	2	2	5			5	2	1		
		carcasses		2	2	1	1		2	1	1	1	4	1			5	3		1	4			1				
		meat							1	6			2								5	2	1	1				
	Human				1				1			1	1				2				4			1	1			
CIP+STR+TET (*n* = 13)	Chicken feces							2		7					1												1	1
	Human									1																		
CIP+NAL+TET+ERY (*n* = 3)	Chicken carcasses				1																							
	Chicken meat		1															1										
CIP+NAL+STR+TET+ERY (*n* = 2)	Chicken feces																			1								
	Human																			1								


CIP, ciprofloxacin; NAL, nalidixic acid; STR, streptomycin; TET, tetracycline; ERY, erythromycin. *New allele not present in the pubMLST database.

### Multidrug Resistance and *flaA*-SVR Subtypes

All 110 multiresistant isolates were classified into 26 different *flaA*-SVR sequence subtypes, mainly 287 (18; 16.4% isolates), 100 (13; 11.8%), and 34 (9; 8.2%) (Table 4). Among 13 *C. jejuni* resistant to CIP, STR and TET two new allele types (1662 and 1663) found in the isolates from chicken feces were identified for the first time and submitted to the pubMLST database.

## Discussion

During the present study a significant *flaA*-SVR diversity among 602 *C. jejuni* isolated from the chicken food chain and from humans with diarrhea was identified. The isolates were collected during a broad range of time (2011–2017) and were obtained in 15 and 5 of 16 voivodeships (administrative regions) of Poland in case of chicken and human *C. jejuni*, respectively. Such representative material may reflect the prevalence and characteristics of the *C. jejuni* isolates all over the whole country. The large numbers of sequence profiles generated may be due to the high variability of the *Campylobacter* genome caused by its instability (Wittwer et al., 2005). It has been previously shown that the *flaA* flagellar gene undergoes spontaneous mutations during the host infection that may play an important role in molecular variation (Guerry, 2007). Among the total of 79 *flaA*-SVR variants, several identical sequences were identified among both human and chicken isolates suggesting a possible chicken source for human infection. Furthermore, on overlap of several genotypes found between chicken isolates recovered from different stages of the food chain may suggest that *C. jejuni* isolates with such allele types are circulating along the chicken meat production chain and may result in transmission of the bacteria to man.

The high genetic diversity of *C. jejuni* tested by the *flaA*-SVR method was previously demonstrated by several authors (Meinersmann et al., 1997; Corcoran et al., 2006; Djordjevic et al., 2007; Wassenaar et al., 2009; Magnússon et al., 2011; Giacomelli et al., 2012; Sing and Kwon, 2013; Gomes et al., 2016). Wassenaar et al. (2009) identified 92 different alleles among 293 *C. jejuni* isolated from three different geographical regions and found that sequence types 36, 32, 34, 15, and 239 were predominant (38.1% of 293 strains tested). Most of these allelic variants (i.e., 36, 34, 15, and 239 were also identified in the present study. Some of these *flaA*-SVR types (e.g., 34 and 36) were previously found in poultry and human *C. jejuni* isolates in Ireland, Italy and Iceland (Corcoran et al., 2006; Magnússon et al., 2011; Giacomelli et al., 2012). It seems that these molecular variants are predominant in Europe and are rarely or never detected in other geographical regions (Sing and Kwon, 2013; Gomes et al., 2016).

Several isolates of chicken and human origins tested in the present study were multiresistant, especially to quinolones, streptomycin and tetracycline. The high potential for resistance to fluoroquinolones in the *Campylobacter* isolates of chicken origin may be associated with the use of these antimicrobials in poultry treatments, although information about antimicrobial usage in the flocks we examined was not available. However, the exceptionally high percentage of *C. jejuni* resistant to quinolones in Poland identified in the present and in previous studies may be due to broad use of these antimicrobials in animal husbandry (Wieczorek et al., 2013, 2015; Woźniak-Biel et al., 2018). According to the recent European Medicines Agency report on fluoroquinolone supply for veterinary medical use, in Poland in 2016 the sales this antimicrobial group (in mg for population correction unit, PCU) were 9.7 mg/PCU, while the average for 30 European countries described in the report in that year was 2.7 mg/PCU (EMA, 2018). Such frequent administration of these drugs may have an influence on the spread of fluoroquinolone-resistant gene determinants in population of these bacteria identified in humans (Aarestrup et al., 2008).

A correlation between specific *flaA*-SVR genotypes and antimicrobial multiresistance among *C. jejuni* tested was not clear and distinct. Isolates with the same resistance pattern were classified into different molecular subtypes whereas the *C. jejuni* with an identical *flaA*-SVR profile were resistant to different antimicrobials. Similarly, other authors likewise found no correlation between genotype and antibiotic resistance (Wittwer et al., 2005; Corcoran et al., 2006). Such difference can be explained by a frequent intra- and interspecies genetic mutation among *C. jejuni* which results with many different molecular variants as determined by the *flaA*-SVR typing. On the other hand, circulation of genetic determinants encoding resistance to more than one antimicrobial may be slower than molecular mutations resulted that such multiresistant isolates are less frequently identified among different *C. jejuni* genotypes (Aarestrup et al., 2008; Iovine, 2013).

In the present study, only a few *C. jejuni* of poultry origin possessed the same multidrug resistance patterns and genotypes as the isolates recovered from humans. This limited correlation may be due to the small number of multiresistant isolates recovered from patients (only 11 isolates) as compared to 99 chicken isolates. Furthermore, it has been shown that such multidrug resistant *C. jejuni* were recovered from patients in only two voivodeships (malopolskie and slaskie) whereas chicken isolates were identified in all over Poland. Therefore, it is difficult to drawn a clear conclusion whether the chicken meat was the source of human multidrug resistant *C. jejuni* infection.

## Conclusion

An important step in control of campylobacteriosis in humans is identification and extensive investigation of *C. jejuni* isolated from the chicken food chain as well as acquisition of full knowledge of their molecular makeup and determination of their resistance to antimicrobials used in treatment of the infection. In the present study a total of 79 different genetic *flaA*-SVR subtypes among 602 isolates were identified which. The obtained results highlighted the lower genetic diversity of human isolates compared with chicken *C. jejuni*. A total of 13 isolates had novel alleles which were not present in the pubMLST database. Some *C. jejuni* tested displayed a multiresistant pattern, mainly to CIP, NAL, STR, and TET and the vast majority of such resistant isolates were of the chicken food chain origin. These *C. jejuni* belonged to 21 different *flaA*-SVR types which shows their broad molecular diversity. Such campylobacters were recovered from the chicken food chain and from patients which may suggest the possible source of human infection.

## Author Contributions

KW and JO contributed to the conception and design of the study, provided samples from the chicken food chain, planned the study, analyzed the data, and drafted the manuscript. TW delivered the human *C. jejuni* isolates. KW and TW performed the experiments. All authors critically read and approved the final version of the manuscript.

## Conflict of Interest Statement

The authors declare that the research was conducted in the absence of any commercial or financial relationships that could be construed as a potential conflict of interest.

## Footnotes

1 http://pubmlst.org/campylobacter/
