# Supplementary material for: flaA-SVR Based Genetic Diversity of Multiresistant Campylobacter jejuni Isolated From Chickens and Humans
Source: Front Microbiol. 2019 May 28;10:1176. doi: 10.3389/fmicb.2019.01176 (PMC6546949; doi:10.3389/fmicb.2019.01176)
Supplement: Supplementary file 2 [file Table_2.DOCX]

**TABLE S2. Antimicrobials, dilution ranges and cut-off values used in the study.**

| Antimicrobial class | Antimicrobial | Dilution range and cut-off value (mg/L) |
| --- | --- | --- |
| Aminoglycosides | Gentamicin (GEN) | 0.12 – 16; >2 |
|  | Streptomycin (STR) | 1 – 16; >4 |
| Macrolides | Erythromycin (ERY) | 0.5 – 32; >4 |
| Quinolones and fluoroquinolones | Ciprofloxacin (CIP) | 0.06 – 4; >0.5 |
|  | Nalidixic acid (NAL) | 2 – 64; >16 |
| Tetracyclines | Tetracycline | 0.25 – 16; >1 |
